# Prognostic impact of psychoeducation program completion on inpatients with schizophrenia: a pilot cohort study

**DOI:** 10.1186/s12888-024-06397-5

**Published:** 2025-01-17

**Authors:** Hiroki Noguchi, Seiichiro Tarutani, Yoshiki Takei, Koichi Matsumoto, Takehiko Okamura, Hiroshi Yoneda

**Affiliations:** 1 Department of Nursing, Shin-Abuyama Hospital, Osaka Institute of Clinical Psychiatry, Takatsuki, 569-1041 Japan; 2 Department of Psychiatry, Shin-Abuyama Hospital, Osaka Institute of Clinical Psychiatry, Takatsuki, 569-1041 Japan; 3 Department of Neuropsychiatry, Osaka Medical and Pharmaceutical University, Takatsuki, 569-8686 Japan

**Keywords:** Schizophrenia, Psychoeducation, Completion, Duration of outpatient treatment

## Abstract

**Background:**

Psychoeducation programs can reduce the risk of recurrence and readmission in patients with schizophrenia. However, almost all previous studies of program efficacy have included only patients completing the program, which may not be possible in all cases. The objective of this pilot cohort study was to compare the prognoses of inpatients with schizophrenia who did or did not complete a well-established institutional psychoeducation program.

**Methods:**

This study is a pilot cohort study, and the participants were 32 inpatients in the psychiatric acute care ward. Among these patients, 18 completed the institutional psychoeducation program by discharge, whereas 14 missed one or more sessions for various reasons. The primary outcome was the duration of outpatient treatment (DOT) during the 5-year follow-up period, and the secondary outcomes were comparisons of the risk of all-cause discontinuation for outpatient treatment and correlations between the program participation rates and DOT.

**Results:**

DOT was significantly longer in the program completion group than in the noncompletion group (918.2 (174.3) days vs. 225.5 (35.7) days, *p* = 0.001), and multivariate Cox proportional hazards regression analysis revealed that program noncompliance was associated with a 4.450-fold (*p* = 0.002) greater risk of discontinuation of outpatient treatment according to multivariate analysis. A significant weak correlation was found for DOT and rates of sessions admitted to the programme (Pearson's *r* = 0.384, *p* = 0.030).

**Conclusions:**

Completion of a psychoeducation program could enhanced the success of outpatient treatment. As psychoeducation and related factors may have a positive effect of the prognosis after discharge, inpatient psychoeducation programs should be flexible enough to provide opportunities for completion.

## Introduction

Schizophrenia is a severe chronic mental disorder characterized by heterogeneous clusters of behavioral, emotional, and cognitive symptoms that are frequently intractable to current psychiatric therapies. As a result, patients require long-term monitoring and pharmacotherapy, including antipsychotic drugs, to improve symptoms and prevent recurrence. In addition, outpatients with schizophrenia experience many additional life challenges, including poor family support, problems with interpersonal relationships, difficulty finding employment, poverty, and side effects of medication that lead to poor treatment adherence [1]. Inpatient care and outpatient management must include interventions that can improve general social functioning. Several studies have reported that inpatients receiving psychoeducation demonstrate better treatment adherence and lower recurrence rates in the short-term and medium- to long-term [2, 3].

Therefore, greater access to effective psychoeducation programs may reduce readmission rates, improve outpatient quality of life (QOL), and lessen the burden on patients with schizophrenia.

However, almost all previous studies on the efficacy of psychoeducation have included only patients completing the program; thus, how noncompletion affects patient prognosis is currently unknown. It has also proven difficult in such studies to closely monitor changes in the psychiatric condition of discharged patients and evaluate the ways in which psychoeducation is used in daily life. Moreover, concrete and common criteria for recurrence or readmission have not been specified in many previous studies [4, 5], but interruptions in social life, including hospitalization, may be traumatic regardless of the reason.

Shin-Abuyama hospital offers a psychoeducation program for inpatients aimed at preventing recurrence and readmission for schizophrenia, similar to many other psychiatric institutions. However, in the real-world clinical setting, unlike in research fields, a certain percentage (sometimes approximately half) of patients were unable to complete the program owing to early discharge or other treatment schedules.

Therefore, it is an urgent task to clarify how the completion or noncompletion of psychoeducation programs affects the long-term prognosis of patients, but to the best of our knowledge, there are no such previous studies.

Thus, this study aimed to clarify how the completion or noncompletion of a psychoeducation program affects all-cause discontinuation in outpatient treatment over a 5-year follow-up period after discharge via a single-center pilot cohort study.

## Methods

### Study design

This is a pilot prospective observational cohort study conducted at the psychiatric acute care ward of Shin-Abuyama Hospital, Osaka Institute of Clinical Psychiatry, Osaka, Japan, from 1st August 2016 to 31st October 2023. The inclusion period of the study was from 1st August 2016 to 31st July 2017 and the follow-up period was set at five years after discharge.

### Selection of study participants

Study participants were recruited as follows. First, potential participants were prescreened by two or more registered nurses responsible for administering the psychoeducation program. Second, among the prescreened patients, the attending psychiatrist made the final decision about participation according to the following criteria: 1) a low risk of self-harm or harm due to psychiatric symptoms; 2) sufficient verbal communication skills; and 3) the ability to pay attention during the 60-min session.

Finally, eligible participants were screened from among the program participants who met the following inclusion criteria: 1) had a diagnosis of schizophrenia (F20) according to the International Statistical Classification of Diseases and Related Health Problems, Tenth Edition (ICD-10) [6]; 2) were admitted to the acute psychiatric ward of Shin-Abuyama Hospital during the inclusion period; 3) participated in a psychoeducation program for inpatients from the first session; and 4) were able to provide informed consent.

The exclusion criteria were as follows: 1) diagnosis of a cooccurring psychiatric disorder, 2) never attended a psychoeducational session, 3) failure to be discharged after the program, or 4) withdrawal of consent.

We defined participants who attended all sessions as the completion group (CG) and those who missed one or more, albeit not all, sessions as the noncompletion group (NG).

### Psychoeducation program for inpatients

The psychoeducation program for inpatients at Shin-Abuyama Hospital consists of five semistructured group sessions (Table 1) based on the Japanese Psychoeducation Promotion Guidelines toolkit [7, 8]. All five sessions were set up by different experts, and the importance of different interventions was the focus of the lectures. Session 1 was conducted by a psychiatrist, Session 2 by an occupational therapist, Session 3 by a mental health worker, Session 4 by a pharmacist, and Session 5 by a nurse. In addition, two or three nurses attended all the sessions as coleaders.

**Table 1:** Contents of Psychoeducation for inpatients with schiphrenia on Shin-Abuyama Hospital

Goals:	
The aim was to support the promotion of disability acceptance, improvement of medication adherence, and prevention of recurrence by providing information on disability and treatment, and related social resources	
Time:	
60 min / session, 1 session / week, total of 5 sessions	
Group structure:	
A closed group with 3 to 4 inpatients	
Method:	
The medical staff in charge of each session provides information to the participants using textbooks, and promotes the sharing of the participants' impressions and their own experiences	
Constitution:	
1. Lecture of schizophrenia (by psychiatrist)	
2. Lecture of occupational therapy (by occupational therapitst)	
3. Introduction to social resources (by mental health worker)	
4. Lecture of medications (by pharmacist)	
5. Lecture of Coping to stress (by nurse)	

If inpatients with schizophrenia did not attend the session, the reasons (worsening of the medical condition, ward transfer, refusal to participate, training for discharge or discharged) were recorded in their medical record and generally categorized as negative (worsening of the medical condition, ward transfer or refusal to participate) or positive reason (training for discharge or discharged).

### Variables

#### Participant characteristics

Age, sex, housing status (home, institution and homeless), living with family or not, marital status (married, divorced and single), employment status (general employment, disabled employment, in employment training and unemployed), highest level of education (university or graduate school, senior high school and junior high school), age of onset (first episode), duration (years) of illness, type of hospitalization (involuntary admission or not), duration (days) of hospitalization in the psychiatric acute care ward, chlorpromazine equivalent dose of antipsychotic medication [9], the rates occupational therapy sessions attended, active participation in occupational therapy sessions (cut-off ≥ 50% or not), the rates of psychoeducation sessions attended.

### Outcome

The primary outcome in this study was the duration of outpatient treatment (DOT) [10]. We defined the day of discharge as day 0 and the day of all-cause discontinuation of outpatient treatment (e.g., readmission, recurrence, suicide, or interruption of regular outpatient hospital visits) as the DOT end day. All participants were followed up for 5 years (1825 days) after discharge via medical records and, if there was difficulty, by phone. In cases where the defining event for DOT could not be determined precisely, we defined the day after the last outpatient visit as censoring and used it for the analysis.

The secondary outcomes were 1) comparative risk (hazard ratio) of all-cause discontinuation of outpatient treatment, 2) the proportion of events precipitating DOT ending each year after discharge, 3) the correlation of DOT with the rates of psychoeducation sessions attended, 4) changes in Global Assessment of Functioning (GAF) scores [11, 12] and QOL dimension scores after the program compared with baseline. GAF scores were collected from medical records. And we adopted the Japanese version of the Schizophrenia Quality of Life Scale (J-SQLS) as a self-rating index of QOL [13, 14]. J-SQLS is a disease-specific subjective QOL rating scale used here as an alternative index of the effect of inpatient treatment. The scale includes 30 items in total with each item scored from 0 to 4. The lower the score, the better the condition. And this scale consists of three subscales: “motivation/energy” (ME), “psychological/social relations” (PS), and “symptoms/side effects” (SS); ME (7 items) assesses motivation and activity levels such as "like to plan ahead", "tend to stay at home and do not go out" and "able to catty out daily activities”. PS (15 items) assesses psychological aspects, including feelings of loneliness, anxiety and depression such as "worry about thing", "feel lonely" and "feel people avoid me". SS (8 items) assesses issues related to medication side effects characteristic of schizophrenia, such as "sleep is disturbed", "get muscle twitches" and "get dizzy spells". J-SQLS was administered both before and after the psychoeducation program, as well as changes in subscores after the intervention, were compared between the CG and NG. And 5) Comparison with CG on different reasons (positive or negative) for NG.

### Study size calculations

Since no previous studies exist, hazard ratios were assumed, and sample sizes were calculated on the basis of clinical realities. A hazard ratio of 0.3 between the CG and NG, an allocation ratio of 1:1, an inclusion period of 1 year, a follow-up period of 5 years, a log-rank test, 80% statistical power, and a type I error rate of *p* = 0.05 were adopted, and the sample size was calculated to be 14 participants per group, for a total of 28 participants [15, 16]. To eliminate the possibility of sampling bias, we attempted to recruit as many participants as possible within the inclusion period.

### Statistical analysis

Statistical analyses were performed via SPSS Statistics version 27 (IBM Corp., Armonk, NY, USA). Patient age, age of onset (first episode), duration (years) of illness, duration (days) of hospitalization in the psychiatric acute care ward, chlorpromazine equivalent dose in the psychiatric acute care ward, the rates of occupational therapy sessions attended, the rates of psychoeducation sessions attended, GAF score, and J-SQLS score were compared between groups by Student’s t test or Welch’s t test, as indicated, after verifying homoscedasticity. The proportions of sex, housing status, living with family or not and type of hospitalization (involuntary admission or not) were compared via Fisher’s exact test. The proportions of marital status, employment status and highest level of education were compared via Fisher-Freeman-Halton’s exact test.

DOT was analyzed via the Kaplan–Meier method and compared between groups via the log-rank test. The hazard ratio for the differential risk of discontinuation of outpatient treatment during follow-up was calculated via the multivariate Cox proportional hazard regression model. To reveal how responsiveness to pharmacotherapy and differences between psychoeducation and treatment attitudes affect prognosis, we selected the following independent variables: duration (days) of hospitalization in the psychiatric acute care ward, chlorpromazine equivalent dose, the rates of occupational therapy session attended and psychoeducation program completion. All these four variables were checked for each interaction using linear multiple regression analysis, and those with no significant interaction were adopted as independent variables for the Cox regression analysis.

Disruption event occurrence rates for each year were compared via Fisher's exact test because fewer than five such events occurred during each year. Correlations between DOT and the rates of sessions attended were analyzed via Pearson’s method.

For the GAF and J-SQLS subscores, comparisons between groups were made using independent t tests for before and after the programme and for the changes.

Of the NG groups, groups were divided into two groups for this reason of NG (positive or negative) and between groups comparisons were performed to reveal the difference of effect to prognosis. And we adopted to compare Mann–Whitney u test for quantitative date and Fisher’s exact test or Fisher-Freeman-Halton’s exact test for categorical data. DOT was analyzed via the Kaplan–Meier method and compared between groups (positive or negative reason) via the log-rank test.

All the statistical comparisons were two-tailed, and the statistical significance level was set at *p* = 0.05. In the case of missing values, those values were excluded, and only the obtained data were analyzed.

### Ethical considerations

This study was conducted with the approval of the Institutional Review Board of Shin-Abuyama Hospital (2016–1) and conformed to the requirements of the latest version of the Declaration of Helsinki. The following ethical considerations were incorporated into the study design, enrollment criteria, follow-up, and analysis: primacy of individual patient wishes, guarantee against therapeutic disadvantages, freedom to withdraw consent, protection of personal data, purpose of use, and disposal of personal data. All participants provided their written consent after receiving a full explanation of the study procedures, long-term follow-up, analysis of participants and patient rights at the time of recruitment.

## Results

### Participant characteristics

**Fig. 1:**
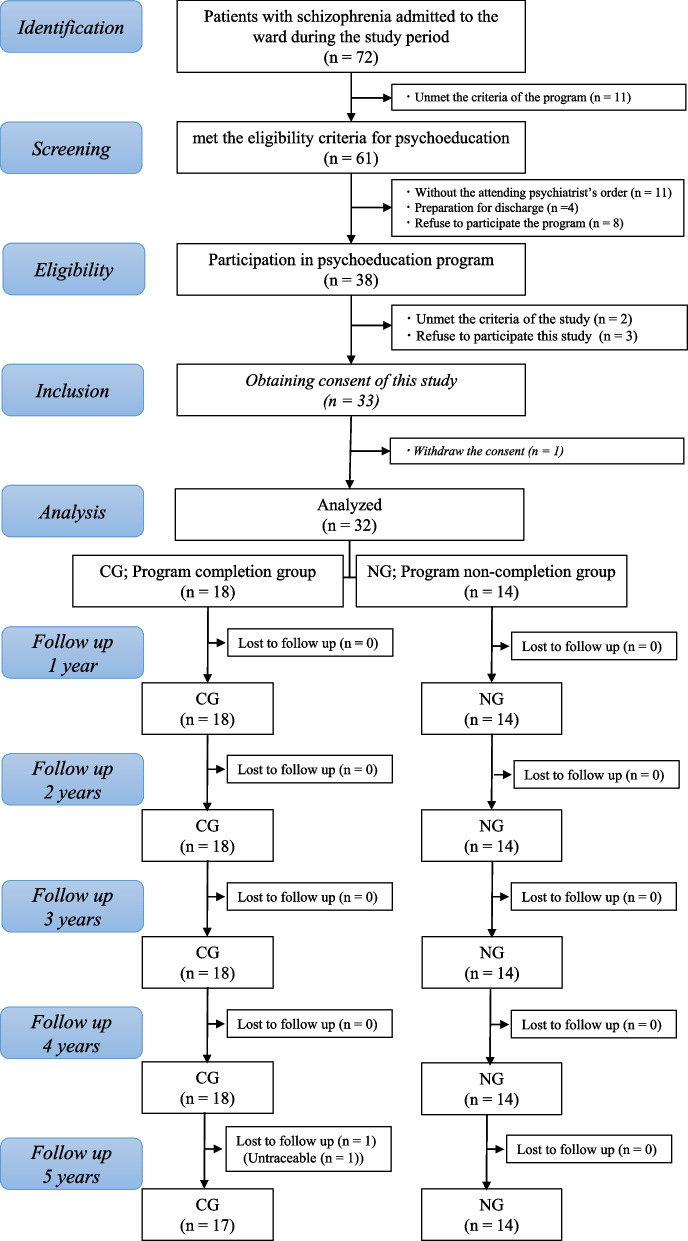
The STROBE study flow chart

A total of 72 patients with schizophrenia were admitted to the ward during the inclusion period, 61 of whom prescreened for psychoeducation program by registered nurses and 50 of whom prescreened by psychiatrist after that. Of these, 38 participated in the program and 36 met the eligibility criteria, 33 of whom consented to the study. Furthermore, of these 33 participants, one was excluded because of withdrawal of consent. Among the 32 eligible participants, 18 were CGs, and 14 were NGs and were followed for up to 5 years. One CG participant was lost to follow-up at day 1543 due to relocation, resulting in a final sample of 17 CG and 14 NG participants (Fig. 1).

Of the 32 inpatients enrolled in the study, 18 completed the psychoeducation program (CG), and 14 did not (NG) (Table 2). There were no significant group differences (CG vs. NG) in age, sex ratio, housing status ratio, living with family ratio, marital status ratio, employment status ratio, highest level of education ratio, age of onset (first episode), duration (years) of illness, type of hospitalization (involuntary admission or not), duration (days) of hospitalization in the psychiatric acute care ward, chlorpromazine equivalent dose, the rates of occupational therapy sessions attended and active participation in occupational therapy sessions or not, except for the rates of psychoeducation session attended (Table 2). Program attendance, the reason for noncompletion, DOT and events by participants are summarized in Table 3. To ensure the anonymity of participants, age is given as age-ranges in Table 3. And all NG groups had an event within the observation period. There were 6 negative reasons for noncompletion (3 worsening the medical condition, 3 ward transfer) and 8 positive reasons (1 discharged, 7 training for discharge), with no obvious “refusals to attend”.

**Table 2:** Comparison of characteristics of the program completion group (CG) and the program noncompletion group (NG)

Characteristics		CG (*n* = 18)		NG (*n* = 14)		*p*
		Mean (S.D) or n (%)		Mean (S.D) or n (%)		
Age		42.7	(9.6)	46	(7.8)	0.299
Sex^a^	Male	6	(33.3)	4	(28.6)	1.000
	Female	12	(66.7)	10	(71.4)	
Housing status^a^	Home	17	(94.4)	13	(92.9)	1.000
	Institution	1	(5.6)	1	(7.1)	
	Homeless	0	(0.0)	0	(0.0)	
Living with family^a^	Yes	13	(72.2)	9	(64.3)	0.712
	No	5	(27.8)	5	(35.7)	
Marital status^b^	Married	3	(16.7)	3	(21.4)	1.000
	Divorced	2	(11.1)	2	(14.3)	
	Single	13	(72.2)	9	(64.3)	
Employment status^b^	General employment	1	(5.6)	1	(7.1)	1.000
	Disabled employment	0	(0.0)	0	(0.0)	
	In employment training	2	(11.1)	2	(14.3)	
	Unemployed	15	(83.3)	11	(78.6)	
Highest level of education^b^	University or Graduate school	2	(11.1)	6	(42.9)	0.139
	Senior high school	11	(61.1)	5	(35.7)	
	Junior high school	4	(22.2)	3	(21.4)	
	Unknown	1	(5.6)	0	(0.0)	
Age of onset (first episode)		25.2	(6.3)	24	(4.9)	0.231
Duration (years) of illness		18.4	(10.6)	23.2	(8.9)	0.178
Involuntary admission^a^		7	(38.9)	8	(87.1)	0.476
Duration (days) of hospitalization in the psychiatric acute care ward^c^		82.8	(13.5)	87.9	(41.0)	0.663
Chlorpromazine equivalent		707.5	(732.1)	530.4	(326.0)	0.407
The rates of occupational therapy sessions attended		52.4	(22.2)	52.9	(30.5)	0.959
Active participation in occupational therapy sessions^a^		10	(55.6)	6	(42.9)	0.722
The rates of psychoeducation sessions attended^c^		100	(0.0)	58.6	(21.4)	< 0.001***

CG: program completion group; NG: program noncompletion group; S.D: standard deviation

^a^: Fisher's exact test was used; ^b^: Fisher-Freeman-Halton's exact test was used

^c^: The Welch's t test was used as used because homoscedasticity could not be confirmed by Levene's test; ***: *p* < 0.001

**Table 3:** List of program attendance for participants in this study

Group	Participants	Age-ranges	Sex	Session 1	Session 2	Session 3	Session 4	Session 5	The reason for noncompletion	Sessions attended the program		DOT^a^ (day)	Event	Cause of events
				Psychiatrist	Occupational therapist	Mental health worker	Pharmacist	Nurse		Number	Rates (%)			
NG (*n* = 14)	1	20–29	F	●	●	-	●	-	Worsening of medical condition	3	60	157	+	Readmission due to reccurence
	2	50–59	F	-	●	●	●	●	Worsening of medical condition	4	80	312	+	Readmission due to reccurence
	3	50–59	F	●	●	●	-	-	Training for discharge	3	60	97	+	Readmission due to reccurence
	4	40–49	F	●	-	●	●	-	Training for discharge	3	60	196	+	Readmission due to reccurence
	5	40–49	F	●	-	●	-	-	Worsening of medical condition	2	40	406	+	Readmission due to reccurence
	6	40–49	M	-	●	-	-	-	Discharged	1	20	507	+	Readmission due to reccurence
	7	50–59	F	●	●	●	●	-	Training for discharge	4	80	93	+	Readmission due to reccurence
	8	40–49	M	●	●	-	●	-	Training for discharge	3	60	209	+	Readmission due to reccurence
	9	40–49	F	-	●	●	●	●	Training for discharge	4	80	107	+	Readmission due to reccurence
	10	50–59	M	-	-	-	-	●	Ward Transfer	1	20	376	+	Readmission due to reccurence
	11	50–59	F	●	-	●	●	-	Ward Transfer	3	60	176	+	Readmission due to reccurence
	12	30–39	F	●	●	-	-	-	Ward Transfer	2	40	199	+	Readmission due to reccurence
	13	40–49	F	●	-	●	●	●	Training for discharge	4	80	273	+	Readmission due to reccurence
	14	40–49	M	●	●	-	●	●	Training for discharge	4	80	49	+	Readmission due to reccurence
CG (*n* = 18)	15	30–39	F	●	●	●	●	●	−	5	100	1548	+	Readmission due to reccurence
	16	30–39	F	●	●	●	●	●	−	5	100	548	+	Readmission due to reccurence
	17	50–59	F	●	●	●	●	●	−	5	100	469	+	Readmission due to reccurence
	18	50–59	F	●	●	●	●	●	−	5	100	1303	+	Readmission due to reccurence
	19	60–69	F	●	●	●	●	●	−	5	100	116	+	Readmission due to reccurence
	20	40–49	F	●	●	●	●	●	−	5	100	162	+	Readmission due to reccurence
	21	40–49	M	●	●	●	●	●	−	5	100	104	+	Readmission due to reccurence
	22	40–49	M	●	●	●	●	●	−	5	100	123	+	Readmission due to reccurence
	23	30–39	F	●	●	●	●	●	−	5	100	1825	-	Right Censoring
	24	20–29	M	●	●	●	●	●	−	5	100	146	+	Readmission due to reccurence
	25	40–49	F	●	●	●	●	●	−	5	100	1825	-	Right Censoring
	26	30–39	M	●	●	●	●	●	−	5	100	1825	-	Right Censoring
	27	50–59	F	●	●	●	●	●	−	5	100	208	+	Readmission due to reccurence
	28	40–49	M	●	●	●	●	●	−	5	100	1825	-	Right Censoring
	29	30–39	F	●	●	●	●	●	−	5	100	1825	-	Right Censoring
	30	50–59	F	●	●	●	●	●	−	5	100	567	+	Right Censoring
	31	30–39	M	●	●	●	●	●	−	5	100	330	+	Right Censoring
	32	30–39	F	●	●	●	●	●	−	5	100	1543	-	Censoring (untraceable)

CG: program completion group; NG: program noncompletion group; DOT: duration of outpatient treatment

^a^: Maximam follow-up period (DOT) in this study is 5 years (1825 days)

### Primary outcome (DOT)

**Fig. 2:**
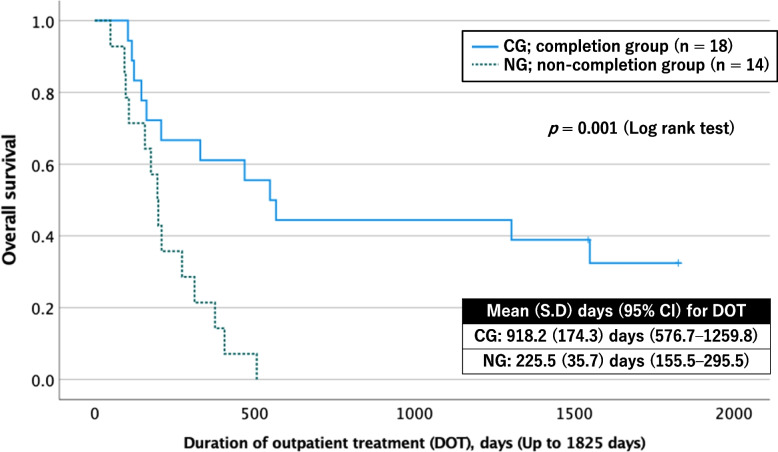
Kaplan–Meier survival curves for the duration of outpatient treatment in the psychoeducation completion (CG) and noncompletion (NG) groups

The final analysis included 18 CG and 14 NG participants. Survival analysis revealed significantly longer DOT in the remaining CG patients than in the remaining NG patients (918.2 (174.3) days, 95% CI: 576.7–1259.8 vs. 225.5 (35.7) days, 95% CI: 155.5–295.5; *p* = 0.001 by log-rank test) (Fig. 2).

### Secondary outcomes

In the linear multiple regression analysis, no significant interactions were found for any combination of the four variables; duration of hospitalization and chrolpromazine equivalent (*p* = 0.978), duration of hospitalization and the rates of occupational therapy sessions attended (*p* = 0.722), duration of hospitalization and psychoeducation program completion (*p* = 0.143), chrolpromazine equivalent and the rates of occupational therapy sessions attended (*p* = 0.753), chrolpromazine equivalent and psychoeducation program completion (*p* = 0.635), and the rates of occupational therapy sessions attended and psychoeducation program completion (*p* = 0.453) (Table 4).

**Table 4:** Multivariate Cox proportional regression analysis of all-cause discontinuation

	HR	95% CI		*p*
		Lower	Upper	
1) Duration (days) of hospitalization	0.992	0.975	1.010	0.395
2) Chrolpromazine equivalent	1.000	0.999	1.001	0.603
3) The rates of occupational therapy sessions attended	0.999	0.980	1,018	0,894
4) Psychoeducation program completion	4.450	1.711	11.574	0.002**

Survival variable: duration of outpatient treatment (DOT), days (up to 1825 days); HR: hazard ratio; **: *p* < 0.01

NOTE: Interactions for each combination by linear multiple regression analysis: 1)*2) (*p* = 0.978), 1)*3) (*p* = 0.722), 1)*4) (*p* = 0.143)

2)*3) (*p* = 0.753), 2)*4) (*p* = 0.635), 3)*4) (*p* = 0.453)

In a multivariate Cox proportional hazard analysis using duration (days) of hospitalization (HR = 0.992, *p* = 0.395), chrolpromazine equivalent (HR = 1.000, *p* = 0.633), the rates of occupational therapy sessions attended (HR = 0,999, *p* = 0894), and psychoeducation program completion as independent variables revealed a significant difference in program completion (HR = 4.450, *p* = 0.002). Participants who did not complete the psychoeducation program were at 4.450-fold greater risk of all-cause discontinuation of outpatient treatment than participants who attended all sessions (Table 4).

For all participants, discontinuation of outpatient treatment was attributed to “readmission due to recurrence” (Table 3). The cumulative number of readmissions was significantly greater among program noncompleters than among program completers by the end of each follow-up year (Table 5).

**Table 5:** Comparison of the percentage of event occurrences by year

	CG (*n* = 18)		NG (*n* = 14)		*p*
	Number	(%)	Number	(%)	
1 year	7	(38.9)	11	(78.6)	0.036*
2 year	10	(55.6)	14	(100)	0.004**
3 year	10	(55.6)	14	(100)	0.004**
4 year	12	(66.6)	14	(100)	0.024*
5 year^a^	12	(70.6)	14	(100)	0.030*

CG: program completion group; NG: program non-completion group; *: *p* < 0.05; **: *p* < 0.01

^a﻿^: 1 participant was censored during the period due to inability to contact. Hence, the population of CG had been reduced to 17

Fisher's exact test was used to compare the percentage of event occurrences by year

**Fig. 3:**
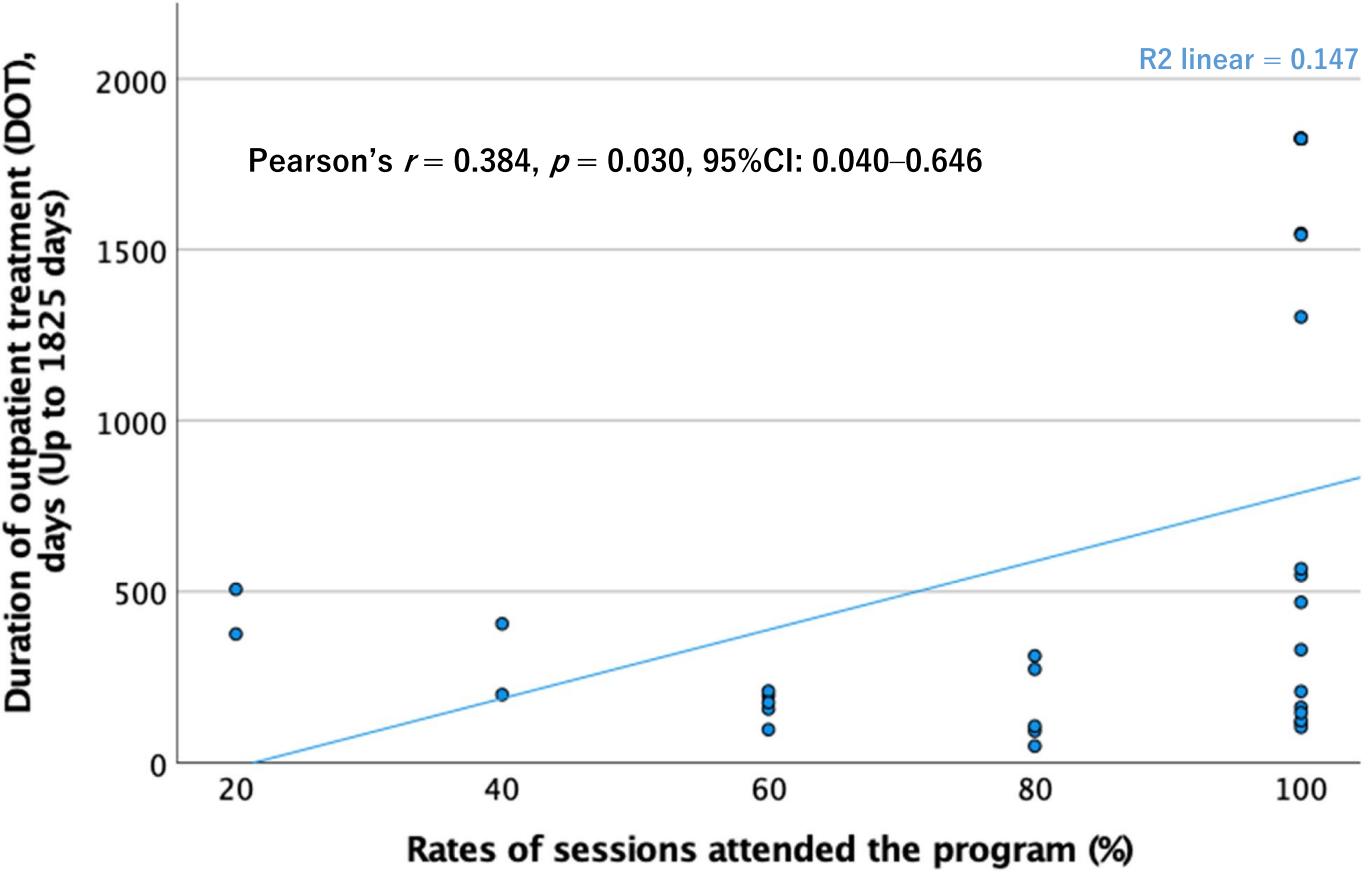
Correlation between DOT and rates of sessions attended the psychoeducation program

There was a significantly weak correlation between DOT and the rates of psychoeducation program sessions attended (Pearson's *r* = 0.384, *p* = 0.030, 95% CI: 0.001–0.646) (Fig. 3).

Between-group comparisons before the program, after the program, and changes in GAF and J-SQLS subscores (ME, PS and SS) revealed no significant differences (Table 6).

**Table 6:** The mean values subscale scores for the completed and non-completed groups of J-SQLS scores

			CG	NG	*p*
			Mean (S.D)	Mean (S.D)	
GAF		*before*	29.8 (11.9)	30.6 (15.9)	0.873
		*after*	58.3 (12.0)	51.6 (12.5)	0.134
		*changes*	28.5 (13.8)	20.6 (16.4)	0.152
J-SQLS	ME	*before*	43.8 (15.7)	44.6 (12.6)	0.877
		*after*	39.7 (19.2)	44.6 (17.7)	0.462
		*changes*	2.5 (15.9)	0.0 (13.9)	0.646
	PS	*before*	46.2 (19.6)	46.9 (17.5)	0.917
		*after*	35.7 (17.9)	36.4 (24.0)	0.923
		*changes*	10.5 (19.3)	10.5 (19.3)	0.998
	SS	*before*	32.1 (13.7)	35.5 (17.0)	0.538
		*after*	26.2 (15.2)	27.2 (15.6)	0.852
		*changes*	5.9 (12.3)	8.3 (15.7)	0.639

CG: program completion group; NG: Program Non-completion group; S.D: standard deviation

ME: motivation/energy; PS: psychological/social relations; SS: symptoms/side effects

In comparison between two groups (positive or negative reason) of NG, no significant group differences were found for all variables (Table 7). And in survival analysis, there were no significant difference between two groups (median 107.0 (70.0) days, 95% CI: 0.0–244.2 vs. median 199.0 (83.3) days, 95% CI: 35.8–362.2; *p* = 0.505 by log-rank test).

**Table 7:** Comparison of characteristics of the reasons (positive or negative reason) in program noncompletion group (NG)

Characteristics		Positive reason (*n* = 8)		Negative reason (*n* = 6)		*p*
		Median (range) or n (%)		Median (range) or n (%)		
Age		46.5	(44–56)	45	(26–56)	0.662
Sex^a^	Male	3	(37.5)	1	(16.7)	0.406
	Female	5	(62.5)	5	(83.3)	
Housing status^a^	Home	8	(100.0)	5	(83.3)	0.429
	Institution	0	(0.0)	1	(16.7)	
	Homeless	0	(0.0)	0	(0.0)	
Living with family^a^	Yes	6	(75.0)	3	(50.0)	0.580
	No	2	(25.0)	3	(50.0)	
Marital status^b^	Married	2	(25.0)	1	(16.7)	1.000
	Divorced	1	(12.5)	1	(16.7)	
	Single	5	(62.5)	4	(66.7)	
Employment status^b^	General employment	1	(12.5)	0	(0.0)	1.000
	Disabled employment	0	(0.0)	0	(0.0)	
	In employment training	1	(12.5)	1	(16.7)	
	Unemployed	6	(75.0)	5	(83.3)	
Highest level of education^b^	University or Graduate school	3	(37.5)	3	(50.0)	1.000
	Senior high school	3	(37.5)	2	(33.3)	
	Junior high school	2	(25.0)	1	(16.7)	
	Unknown	0	(0.0)	0	(0.0)	
Age of onset (first episode)		24	(16–35)	23	(16–35)	0.95
Duration (years) of illness		26.5	(15–33)	21.5	(5–35)	0.662
Involuntary admission^a^		3	(37.5)	5	(83.3)	0.138
Duration (days) of hospitalization in the psychiatric acute care ward^c^		87	(47–138)	81.5	(20–158)	0.852
Chlorpromazine equivalent		387.5	(100–800)	462.5	(100–1400)	0.108
The rates of occupational therapy sessions attended		44.6	(31.3–73.1)	76.3	(0–100)	0.491
Active participation in occupational therapy sessions^a^		2	(25.0)	4	(66.7)	0.277
The rates of psychoeducation sessions attended^c^		70	(20–80)	50	(20–80)	0.181

^a^: Fisher's exact test was used; ^b^: Fisher-Freeman-Halton's exact test was used

^c^The Welch's t test was used as used because homoscedasticity could not be confirmed by Levene's test

## Discussion

To the best of our knowledge, this is the first study comparing the long-term (5 years) efficacy of inpatient psychoeducation for schizophrenia management after hospital release between participants attending all sessions and participants missing one or more (but not all) sessions. Indeed, this is possibly the first pilot cohort study to examine the prognostic impact of the frequency of psychoeducation participation in a small but real-world setting. Therefore, the NG demonstrated an approximately 4.5-fold greater risk of all-cause discontinuation of outpatient treatment as well as earlier readmission for disease relapse. Despite the small sample size in this study, there were no differences in characteristic factors related to schizophrenia treatment in the two groups, including housing, living, marriage, employment and education. Although no significant factors other than psychoeducation completion were found, the possibility cannot be excluded that this may indicate the existence of potential factors affecting psychoeducation completion. On the other hand, no interaction was found for participation in occupational therapy. the results may have been contributed to by the acquisition of knowledge and skills through lectures and/or cognitive-behavioral therapeutic interventions in psychoeducation, rather than occupational therapy. Therefore, it may be difficult to say that, at least, only good (or bad) treatment attitudes have an effect on prognosis. In addition, we have examined the possibility that reasons (positive or negative) for NG might affect prognosis, but it is also clear that noncompletion for positive reasons for treatment does not necessarily predict a positive outcome. Although these may also support the need for psychoeducation program completion, it is essential to consider some potential factors for the reasons leading to completion or noncompletion of psychoeducation and their association with prognosis.

However, although the results of this study suggested that psychoeducation improved long-term outcomes, there were no significant differences between the two groups in QOL or GAF in the short-term. The results of this study are not consistent with those of a previous study in the short-term [17]. A recent meta-analysis concluded that complete psychoeducation reduced the recurrence/readmission rate among patients with schizophrenia spectrum disorders in the acute phase [18]. These interventions had similar preconditions as those of the current study. This discrepancy in results may indicate that this study may have failed to employ appropriate measures of the short-term effects of psychoeducation, as well as missing potential and important factors that influence completion of psychoeducation. Nevertheless, as psychoeducation programs could have a positive impact on prognosis, it will be essential to provide such programs with sufficient flexibility so that they can be completed by all inpatients.

Robinson et al. [19] reported that more than half of patients with schizophrenic disorders (63.1%) relapsed within 3 years after the first hospitalization, a rate similar to that of our CG. In contrast, all patients who did not complete the psychoeducation program relapsed within 2 years of discharge. Patients with poor medication adherence as outpatients are 2.4 times more likely to be rehospitalized than those with good medication adherence, and nonadherence is the major cause of relapse or recurrence [20, 21]. The lower recurrence rate among patients completing our psychoeducation program cannot be explained by the characteristics of the participants in the NG (Table 2); however, the study could suggest that the program may have contributed to some change in life or treatment attitude related to improve medication adherence.

The efficacy of psychoeducation for preventing recurrence has been verified by multiple studies [2, 3, 22]. In addition, psychoeducation was reported to improve patient QOL [23]. Although this study did not reveal direct improvements in QOL, it would be necessary to clarify how the completion of psychoeducation contributed to patients' QOL and other factors that stabilized their lives after discharge.

There are several distinct models of psychoeducation for patients with schizophrenia, such as programs including the participation of families as well as patients [24], programs delivered exclusively for outpatients or continuing in an outpatient setting [25], and community-based programs [23]. Programs adopting a combination of educational, behavioral, and emotional strategies are highly effective at maintaining medication adherence and reducing recurrence and readmission [26], whereas psychoeducational interventions without behavioral elements or support services may not be as effective [27, 28]. In this study, the target, method, frequency, and environment of the intervention differed from those of the aforementioned studies; thus, it is difficult to fully explain the comparison of efficacy rates and the reason for prolonged DOT. Nonetheless, completion of these programs appears essential for full efficacy [17].

The only clear difference between completers and noncompleters is the amount of knowledge acquired [29], but a causal relationship between the amount of knowledge and recurrence risk has not been demonstrated. Similarly, while poor medication adherence is strongly associated with recurrence [30], no causal relationship has been established between the contents of psychoeducation and adherence. The multiaxial approach to psychoeducation programs by five professions (psychiatrists, occupational therapists, mental health workers, pharmacists and nurses) may have influenced the results of this study and further confirmed a possible correlation between the rates of sessions attended and DOT. These findings strongly support the hypothesis that the completion of psychoeducation programs is essential for relapse prevention and suggest that more program attendance improves outpatient outcomes. In the future, expanding the sample size and further analyzing the relationship between missed content and recurrence may be useful in developing and improving psychoeducation programs.

### Limitations

This study is a pilot study; however, owing to the small sample size, a larger (possibly multicenter) study is warranted. Small sample size could be affected sampling bias and lead statistical errors. And this study have some other major limitations.

First, it is the presence of potential confounding factors. We have not been able to adequately identify a range of potential confounding factors related to schizophrenia treatment. And this study could only partially clarify which elements of the patients were affected by psychoeducation program completion and how the reasons for program noncompletion affected their prognosis. It would have been necessary to consider the influence of the patient's characteristics, including their attitude to treatment, and their supportive environment.

Second, It is about the setting of independent variables in multivariate analyses. There are several well-known factors that may affect schizophrenia treatment or prognosis. In this study, we focused on interventions in inpatient treatment that could influence psychoeducation. Due to sample size limitations, we had to give up some important variables. Although there were no obvious differences between the two groups among the basic attribute variables employed in this study, the possibility that the choice of variables may have influenced the results cannot be excluded.

Third, it is differences in delivery among leaders of the psychoeducation program. Although our psychoeducation program was created on the basis of a toolkit, there is no nation-wide program in Japan for training psychoeducation practitioners. There are also major differences in program content and emphasis (e.g., number of sessions) among centers. To solve this problem, establishing a standardized program and supervision system to ensure the homogeneity of the program's effectiveness are essential.

Forth, there may be marked differences in cognitive abilities among patients, further compounding heterogeneity in the outcome within and among treatment centers. Although there was no statistically significant difference in the GAF score between the completion and noncompletion groups, it has been reported that disease severity can hinder program completion. More vulnerable patients may not be able to fully learn and use the coping strategies included in the program to prevent recurrence [31]. Such cases may require more pervasive monitoring rather than relying on the benefits of psychoeducation. It is also critical to identify and validate the most therapeutically effective elements of the program for emphasis, especially for cases with limited cognitive capacity.

Finally, we were not able to verify whether the prognosis improved as a result of the completion of psychoeducation and adherence to medication or whether the prognosis improved as a result of improved attitudes toward medication or insight into the disease due to the completion of psychoeducation. To solve this problem, additional studies that include assessments of personality and cognitive function would be appropriate.

### Future perspective

It has been reported that medication adherence may decrease with time following schizophrenia diagnosis [12], whereas the risk of death may increase [32]. These findings suggest that interventions aimed at improving adherence should instead be instituted or repeated during this critical period. There is also evidence that psychoeducation is not effective for patients at onset [33]. Therefore, first hospitalization is an appropriate time for psychoeducation despite challenges in some cases, such as early release or poor patient condition. Future studies should expand the sample size to examine how program completion is related to DOT, along with attitudes toward medication, insight into the disease and medication adherence.

## Conclusion

Noncompletion of an inpatient psychoeducation program could resulted in a significantly shorter duration of uninterrupted outpatient treatment and earlier symptom recurrence. These results may suggest that the completion of psychoeducation programs and related potential factors have a positive effect on patient prognosis. All efforts should be made to allow inpatients the opportunity to complete psychoeducation programs as early as possible after the onset of illness despite time constraints and other challenges to prevent relapse or recurrence.

## Data Availability

The datasets used and/or analyzed during the current study are available from the corresponding author upon reasonable request.
